# Entry screening to delay local transmission of 2009 pandemic influenza A (H1N1)

**DOI:** 10.1186/1471-2334-10-82

**Published:** 2010-03-30

**Authors:** Benjamin J Cowling, Lincoln LH Lau, Peng Wu, Helen WC Wong, Vicky J Fang, Steven Riley, Hiroshi Nishiura

**Affiliations:** 1 School of Public Health, The University of Hong Kong, Hong Kong Special Administrative Region, People's Republic of China; 2 Imperial College London, London, UK; 3 Theoretical Epidemiology, University of Utrecht, Utrecht, The Netherlands; 4 PRESTO, Japan Science and Technology Agency, Saitama, Japan

## Abstract

**Background:**

After the WHO issued the global alert for 2009 pandemic influenza A (H1N1), many national health agencies began to screen travelers on entry in airports, ports and border crossings to try to delay local transmission.

**Methods:**

We reviewed entry screening policies adopted by different nations and ascertained dates of official report of the first laboratory-confirmed imported H1N1 case and the first laboratory-confirmed untraceable or 'local' H1N1 case.

**Results:**

Implementation of entry screening policies was associated with on average additional 7-12 day delays in local transmission compared to nations that did not implement entry screening, with lower bounds of 95% confidence intervals consistent with no additional delays and upper bounds extending to 20-30 day additional delays.

**Conclusions:**

Entry screening may lead to short-term delays in local transmission of a novel strain of influenza virus. The resources required for implementation should be balanced against the expected benefits of entry screening.

## Background

Pandemic influenza A (H1N1) virus emerged in Mexico in early 2009. Rapid global spread was primarily associated with air travel [1]. As the World Health Organization (WHO) raised their pandemic alert level to 4 and then 5 in April, national health agencies across the world activated their pandemic plans. Following guidance by WHO, many authorities began to screen travelers on entry in airports, ports and border crossings, isolate suspected or confirmed cases, and quarantine their close contacts [2]. Exit screening was not implemented by source nations. Modeling studies suggested that entry screening could not prevent introduction but might be able to delay local epidemics by a few weeks [3-6]. Entry screening and quarantine did not substantially delay introductions in previous pandemics [7]. We reviewed entry screening policies adopted by different nations and estimated the range of delays in local epidemics associated with entry screening.

## Methods

To explore potential delays in local H1N1 transmission associated with entry screening, we ascertained dates of official report of the first laboratory-confirmed imported H1N1 case and the first laboratory-confirmed untraceable or 'local' H1N1 case (i.e. a case not otherwise epidemiologically linked with international travel, contact with an imported case or their secondary infectees) and the interval between these two events. We calculated the additional delays associated with entry screening tools versus the observed delays in nations that did not screen. Since the data did not follow a normal distribution we estimated 95% confidence intervals for these differences using bootstrapping, which is a resampling technique suitable for statistical inference in small sample sizes with non-normal distributions [8]. We based each bootstrap confidence interval on 1,000 resamples. Statistical analyses were conducted using R (R Development Core Team, Vienna, Austria) [9].

The study was conducted between July 13 and August 22, 2009. The methods of entry screening employed were identified by review of official national health ministry websites and the media, and Google searches in English using queries of the form ("<country name>" AND ("influenza" OR "H1N1" OR "swine flu" OR "pandemic" OR "Mexican flu")). We included each nation that had notified more than 100 confirmed H1N1 cases to the World Health Organization by July 6, 2009, except Mexico and the United States where local transmission occurred prior to the WHO global alert. To determine the date of first imported case and first local case the search queries were extended accordingly. Queries were translated by Google language tools http://www.google.com/language_tools and Babelfish http://babelfish.yahoo.com to local official languages and searches were repeated to further increase our scope. We searched for websites in languages including Chinese, Dutch, French, German, Greek, Hebrew, Japanese, Korean, Portuguese, Spanish and Thai.

## Results

We identified 35 nations that had reported more than 100 confirmed H1N1 cases to the World Health Organization by July 6 (we included Hong Kong separately from mainland China as it has separate administration) (Additional file 1). The date of the first untraceable local case could not be determined for 9/35 of the nations. Further details and web links to relevant reports and original data sources are available from the corresponding author on request.

We identified four broad approaches to entry screening. First, temperature checks were performed onboard aircraft prior to disembarkation. Second, health declaration forms were collected from every traveler or all travelers from countries identified with confirmed H1N1 cases. Third, arriving travelers were observed by alert staff for influenza symptoms (e.g. cough). Fourth, travelers were scanned for elevated body temperature by thermal scanners. In the majority of countries screening was implemented by May 1, 2009 although we were unable to determine whether there were any substantial changes in screening protocols after commencement of screening but before confirmation of the first local case.

Because of stochasticity (i.e. chance variations in the occurrence of secondary transmission due to small number of cases initially), the single observed interval between the confirmation of the first imported case and the first local case in a given country is not easily interpretable. We examined patterns in aggregated data expecting that errors due to stochasticity should tend to average out in comparisons between groups of countries using similar tools. Two nations (China and Japan) implemented all four tools. Five nations did not implement any of the four. Table 1 shows the intervals between confirmations of first imported cases and first local cases, categorized by entry screening tools. Overall, implementation of the four tools alone or in combination were associated with on average additional 7-12 day delays in local transmission compared to nations that did not implement entry screening, with lower bounds of 95% confidence intervals consistent with no additional delays and upper bounds extending to 20-30 day additional delays (Table 1). Dates of illness onset were available for the first imported cases in 11/26 nations and the first local cases in 4/26 nations, and mean delays were similar in that subset (data not shown).

**Table 1:** Use of alternative entry screening approaches and intervals between official confirmation of first imported pandemic influenza A (H1N1) case and official confirmation of first untraceable local case for 26 nations with more than 100 confirmed cases by July 6, 2009.

Screening approaches used	n (%)	Median interval, days (inter-quartile range)	Mean interval, days	Mean difference in intervals compared to no screening (95% CI)*
No screening	5 (19%)	22 (0, 22)	14	
1- Medical checks before disembarkation	2 (8%)	21 (14, 28)	21	7 (-14, 30)
2- Health declaration forms	11 (42%)	22 (13, 34)	23	9 (-4, 24)
3- Symptom screening	13 (50%)	33 (7, 41)	26	12 (-2, 27)
4- Thermal scanners	13 (50%)	22 (7, 33)	21	7 (-6, 23)
2 OR 3 OR 4	21 (81%)	22 (7, 35)	23	9 (-3, 22)
2 AND 3 AND 4	6 (23%)	23 (9, 35)	22	7 (-9, 25)

*95% confidence intervals estimated by bootstrapping with 1,000 resamples.

## Discussion

Our results suggest that entry screening did not lead to substantial delays in local H1N1 transmission (Table 1). This empirical study is consistent with theoretical results from previous modeling studies [3-6] and findings from previous pandemics [7]. While longer delays in local transmission to the summer in countries in the Northern hemisphere could have substantially aided pandemic mitigation, due to seasonal factors [3] and school vacations [10,11] leading to lower peak attack rates [12], the observed delays in the present pandemic suggest entry screening provided around 1-2 weeks of additional time for preparation and planning.

While our study focused on the impact of entry screening, some nations also implemented other containment and mitigation measures, such as isolation of suspected or confirmed cases, quarantine of their contacts with or without antiviral chemoprophylaxis, school closures or other social distancing measures, and public health campaigns to improve hygiene. Most nations enhanced their influenza surveillance. If countries that expended greater effort into entry screening also had more effective containment and mitigation measures in the general population, these might have led us to overestimate the effect of entry screening. Conversely, if countries that expanded greater effort into entry screening also tended to have better influenza surveillance and were able to identify local transmission earlier, we may have underestimated the effect of entry screening. Other differences between countries in laboratory capacity and availability of public health resources may also have confounded our evaluation, and all of these factors are limitations of our study.

Previous mathematical modeling studies have questioned the value of entry screening, since it could only delay rather than prevent local epidemics [3-6]. However, most models assumed that source countries would conduct exit screening and infectious cases would not travel [3-6]. In such a scenario it is not surprising that entry screening would add little benefit, since most journeys are shorter than the average 1.5-2 day incubation period for influenza A virus infections [5,13]. Screening is unlikely to identify 100% of ill travelers, while some might use antipyretics to reduce a fever prior to passing through thermal scanners, or fail to report symptoms on declaration forms. Many individuals with subclinical or asymptomatic illness would not be identified, and could initiate outbreaks after arrival [14]. In Hong Kong, only one third of confirmed imported H1N1 cases were identified through screening on entry to Hong Kong, the majority of imported cases were identified through the local health care system after arrival (T. Tsang, personal communication). A similar experience has been reported in Singapore [15]. Nevertheless, entry screening could act as a deterrent to traveling when ill or lead to other indirect benefits such as improving public awareness of the pandemic.

For entry screening to be successfully employed, substantial resources are required to identify the small fraction of travelers who may have H1N1 infection [16]. Further resources may be needed to isolate identified cases, and trace and quarantine close contacts. An important caveat is that a delay in inevitable local transmission of a pandemic virus may not be desirable if it would defer local transmission into a season associated with higher transmissibility such as the winter in temperate regions [12], or if it led to importation and local transmission of antiviral resistant strains [17].

In addition to the caveats on potential confounding by resource availability, competing interventions, and other differences discussed above, there are a number of further limitations to our study. First, identification, confirmation and notification of H1N1 cases is unlikely to have been perfect given the mild and self-limiting nature of most infections, and dates of importation and local transmission that we report may lag behind the true events of interest. Nations that devoted greater resources to entry screening may have identified imported cases earlier. Secondly, we have not considered the size of local epidemics, or how the degree of connectivity with source regions (for example the number of travelers per day) might relate to time delays between imported and local cases. Thirdly, by focusing on nations with at least 100 confirmed cases by July 6, 2009 we may have excluded nations where entry screening was more effective in delaying local transmission, or excluded some nations with fewer resources available for surveillance and confirmation of local cases. Fourthly, while we searched for the dates of reporting of the first imported case and first local case, these dates may not have corresponded exactly to the dates of identification and confirmation of those cases, since in some cases delays may have occurred for various reasons including political considerations. Finally, we collected data from online sources including official government websites, and we have included the hyperlinks in Additional file 1, but information available on the internet could be inaccurate.

## Conclusions

In conclusion, our results suggest that entry screening could delay local transmission for an additional 1-2 weeks. The uncertainty bound of the delay estimates ranged from no delay to 20-30 days delay. A delay of 1-2 weeks could be useful if the additional time permits more comprehensive planning and preparation for a local epidemic, or shortens the time required for other pandemic mitigation measures such as school closures to be sustained. However the benefits of local screening should be balanced against the considerable resources required to implement screening [14]. Our empirical results are consistent with the modeling literature, and support the guidance from the World Health Organization that entry screening can only prevent local spread for a short period of time [14].

## Competing interests

The authors declare that they have no competing interests.

## Authors' contributions

BJC conceived of the study and drafted the manuscript. BJC, LLH, PW and HWCW participated in data collection. VJF conducted the statistical analyses. SR participated in interpreting the results. HN participated in planning the study and interpreting the results. All authors were involved in critical review and editing of the first draft, and subsequent revisions to the manuscript. All authors read and approved the final manuscript.

## Pre-publication history

The pre-publication history for this paper can be accessed here:

http://www.biomedcentral.com/1471-2334/10/82/prepub

## Supplementary Material

Additional file 1 Use of entry screening* and interval between confirmation of first imported pandemic influenza A (H1N1) case and confirmation of first untraceable local case Click here for file
