# Supplementary material for: Entry screening to delay local transmission of 2009 pandemic influenza A (H1N1)
Source: BMC Infect Dis. 2010 Mar 30;10:82. doi: 10.1186/1471-2334-10-82 (PMC3152767; doi:10.1186/1471-2334-10-82)
Supplement: Additional file 1 — Use of entry screening* and interval between confirmation of first imported pandemic influenza A (H1N1) case and confirmation of first untraceable local case [file 1471-2334-10-82-S1.DOC]

Additional file 1. Use of entry screening* and interval between confirmation of first imported pandemic influenza A (H1N1) case and confirmation of first untraceable local case

| **Nation** | **Confirmed cases by July 6** | **1. Medical checks** | **2. Health declaration forms** | **3. Symptom screening** | **4. Thermal scanners** | **First imported case** | **First untraced case** | **Interval, days** |
| --- | --- | --- | --- | --- | --- | --- | --- | --- |
| Canada | 7983 | - | [Y](http://blog.taragana.com/n/canada-tightens-airport-screening-for-swine-flu-41656/) | - | - | [26 April](http://www.phac-aspc.gc.ca/media/nr-rp/2009/2009_0426_h1n1-eng.php) | [9 May](http://albertatalks.ca/2009/05/09/four-additional-h1n1swine-flu-cases-reported-in-alberta/) | 13 |
| United Kingdom | 7447 | - | - | [Y](http://www.fco.gov.uk/en/travelling-and-living-overseas/swine-flu) | - | [27 April](http://www.dh.gov.uk/en/News/Recentstories/DH_098574) | [1 May](http://www.dh.gov.uk/en/Publichealth/Flu/Swineflu/News/DH_101986) | 4 |
| Chile | 7376 | - | [Y](http://www.redsalud.gov.cl/noticias/noticias.php?id_n=437&show=8-2009) | [Y](http://news.bbc.co.uk/2/hi/americas/8022516.stm) | [Y](http://www6.miami.edu/UMH/CDA/UMH_Main/1,1770,6371-1%3B66336-3,00.html) | [17 May](http://new.paho.org/hq/index.php?option=com_content&task=view&id=1377&Itemid=1206) | [18 May](http://www.santiagotimes.cl/santiagotimes/index.php/2009051816287/news/health-science-news/three-more-cases-of-swine-flu-confirmed-in-chile.html) | 1 |
| Australia | 5298 | - | [Y](http://www.healthemergency.gov.au/internet/healthemergency/publishing.nsf/Content/news-008) | [Y](http://www.healthemergency.gov.au/internet/healthemergency/publishing.nsf/Content/news-001) | [Y](http://www.healthemergency.gov.au/internet/healthemergency/publishing.nsf/Content/news-008) | [9 May](http://www.healthemergency.gov.au/internet/healthemergency/publishing.nsf/Content/news-012) | [22 May](http://www.healthemergency.gov.au/internet/healthemergency/publishing.nsf/Content/news-020) | 13 |
| Argentina | 2485 | - | [Y](http://www.fco.gov.uk/en/travelling-and-living-overseas/travel-advice-by-country/south-america/argentina) | [Y](http://www.fco.gov.uk/en/travelling-and-living-overseas/travel-advice-by-country/south-america/argentina) | - | [8 May](http://new.paho.org/hq/index.php?option=com_content&task=view&id=1329&Itemid=1206) | ? |  |
| Thailand | 2076 | - | - | - | [Y](http://beid.ddc.moph.go.th/eng/images/stories/SwineFlu/Ministry of Public Health 7 .52.doc) | [12 May](http://beid.ddc.moph.go.th/th/images/news/mophadvice5eng.doc) | [10 June](http://www.nationmultimedia.com/search/read.php?newsid=30104804&keyword=h1n1) | 29 |
| Japan | 1790 | [Y](http://www.internationalsos.com/PandemicPreparedness/SubCatLevel.aspx?li=6&languageID=ENG&subCatID=87) | [Y](http://www.washingtonpost.com/wp-dyn/content/article/2009/05/04/AR2009050400688.html) | [Y](http://wwwn.cdc.gov/travel/content/news-announcements/delays-H1N1-screening.aspx) | [Y](http://search.japantimes.co.jp/cgi-bin/nn20090510a2.html) | [9 May](http://idsc.nih.go.jp/disease/swine_influenza_e/idsc_e2009/09idsc1e.html) | [16 May](http://idsc.nih.go.jp/disease/swine_influenza_e/idsc_e2009/09idsc5e.html) | 7 |
| Philippines | 1709 | - | [Y](http://www.doh.gov.ph/node/2258) | - | [Y](http://www.doh.gov.ph/node/2258) | [21 May](http://www.doh.gov.ph/h1n1/index.php?option=com_content&view=article&id=84:update-no-18-rps-1st-h1n1-case&catid=35:daily-influenza-a-h1n1-updates&Itemid=61) | [15 June](http://www.doh.gov.ph/h1n1/index.php?option=com_content&view=article&id=112:update-no-41-duque-reports-33-more-ah1n1-cases-sent-home-over-the-week-end-while-confirming-community-outbreak-in-one-barangay-in-jaen-&catid=35:daily-influenza-a-h1n1-updates&Itemid=61) | 25 |
| New Zealand | 1059 | - | [Y](http://www.controlrisks.com/default.aspx?page=1444) | [Y](http://www.controlrisks.com/default.aspx?page=1444) | - | [28 April](http://www.moh.govt.nz/moh.nsf/indexmh/results-of-h1n1-swine-flu-testing-280409) | [13 June](http://www.moh.govt.nz/moh.nsf/indexmh/influenza-a-h1n1-update-seventytwo-130609) | 46 |
| Singapore | 1055 | - | [Y](http://www.moh.gov.sg/mohcorp/uploadedFiles/Web_Parts/swineflu/Statement by Health Minister _12 May_.pdf) | - | [Y](http://www.moh.gov.sg/mohcorp/pressreleases.aspx?id=21508) | [27 May](http://www.moh.gov.sg/mohcorp/pressreleases.aspx?id=21914) | [18 June](http://www.moh.gov.sg/mohcorp/pressreleases.aspx?id=22194) | 22 |
| China^†^ | 1007 | [Y](http://www.moh.gov.cn/publicfiles/business/htmlfiles/mohbgt/s3582/200905/40498.htm) | [Y](http://www.moh.gov.cn/publicfiles/business/htmlfiles/mohbgt/s3582/200905/40498.htm) | [Y](http://www.moh.gov.cn/publicfiles/business/htmlfiles/mohbgt/s3582/200905/40498.htm) | [Y](http://www.moh.gov.cn/publicfiles/business/htmlfiles/mohbgt/s3582/200905/40498.htm) | [11 May](http://www.chinacdc.net.cn/n272442/n272530/n273736/n273781/n4624704/n4624712/31084.html) | [15 June](http://www.gdemo.gov.cn/zt/zlg/dt/gd/200906/t20090614_95058.htm) | 35 |
| Hong Kong | 973 | - | [Y](http://www.chp.gov.hk/content.asp?lang=en&info_id=16770&id=116) | [Y](http://www.chp.gov.hk/content.asp?lang=en&info_id=16626&id=116) | [Y](http://www.fhb.gov.hk/en/press_and_publications/press/2009/press090426a.htm) | [1 May](http://www.info.gov.hk/gia/general/200905/02/P200905020014.htm) | [11 June](http://www.info.gov.hk/gia/general/200906/11/P200906110198.htm) | 41 |
| Peru | 916 | - | - | - | - | [14 May](http://www.dge.gob.pe/boletines/2009/22.pdf) | [5 June](http://www.dge.gob.pe/influenza/AH1N1/sala/Sala_pandemia_05-06-2009.pdf) | 22 |
| Spain | 776 | - | - | [Y](http://www.elmundo.es/elmundo/2009/04/27/espana/1240832632.html) | - | [27 April](http://72.14.203.132/translate_c?hl=en&sl=es&u=http://www.msc.es/en/gabinetePrensa/notaPrensa/desarrolloNotaPrensa.jsp%3Fid%3D1473&prev=/search%3Fq%3Dspain%2Bministry%2Bof%2Bhealth%26hl%3Den%26client%3Dfirefox-a%26rls%3Dorg.mozilla:en-US:official%26hs%3DDD5&rurl=translate.google.com&usg=ALkJrhi4N03q90Vw7OCkaGDHsP-SlvSgXA) | [4 May](http://www.hanken.fi/media/2109/ecdcsituationreport090504.pdf) | 7 |
| Brazil | 737 | - | [Y](http://www.controlrisks.com/default.aspx?page=1444) | - | [Y](http://www.meditherm.com/assets/Meditherm news release 04.30 (1).doc) | [7 May](http://translate.google.com/translate?hl=en&sl=pt&tl=en&u=http%3A%2F%2Fportal.saude.gov.br%2Fportal%2Faplicacoes%2Fnoticias%2Fdefault.cfm%3Fpg%3DdspDetalheNoticia%26id_area%3D124%26CO_NOTICIA%3D10169) | ? |  |
| Israel | 681 | - | - | [Y](http://www.centreforaviation.com/news/2009/05/12/confirmed-swine-flu-cases-still-growing-24-countries-now-affected---latest-aviation-updates/page1) | [Y](http://www.meditherm.com/assets/Meditherm news release 04.30 (1).doc) | [28 April](http://www.euro.who.int/influenza/AH1N1/20090428_7) | [4 June](http://www.ynetnews.com/articles/0,7340,L-3726149,00.html) | 37 |
| Germany | 505 | - | - | [Y](http://www.rki.de/cln_160/nn_200132/DE/Content/InfAZ/I/Influenza/influenzapandemieplan__I,templateId=raw,property=publicationFile.pdf/influenzapandemieplan_I.pdf) | - | [29 April](http://www.rki.de/cln_162/nn_200120/DE/Content/Service/Presse/Pressemitteilungen/2009/08__2009.html) | [16 June](http://www.eurosurveillance.org/ViewArticle.aspx?ArticleId=19295) | 48 |
| Panama | 417 | - | - | - | [Y](http://www.laestrella.com.pa/mensual/2009/05/16/contenido/99468.asp) | [9 May](http://www.searo.who.int/LinkFiles/Influenza_A(H1N1)_Chronology_of_Influenza_A(H1N1).pdf) | ? |  |
| Bolivia | 416 | - | [Y](http://abi.bo/index.php?i=noticias_texto&j=20090518150624lx) | - | - | [29 May](http://abi.bo/index.php?i=noticias_texto&j=20090529193444lx) | [17 June](http://abi.bo/index.php?i=noticias_texto&j=20090617104747lx) | 19 |
| Nicaragua | 321 | - | - | - | - | [2 June](http://new.paho.org/hq/index.php?option=com_content&task=view&id=1455&Itemid=1206) | [2 June](http://www.internationalsos.com/pandemicpreparedness/CountryLevel.aspx?languageID=ENG&countryID=32&catID=27) | 0 |
| El Salvador | 319 | - | - | - | [Y](http://www.mspas.gob.sv/comunicaciones/archivos_comunicados2009/comunicado30042009.asp) | [3 May](http://new.paho.org/hq/index.php?option=com_content&task=view&id=1298&Itemid=1206) | [18 May](http://www.elsalvador.com/mwedh/nota/nota_completa.asp?idCat=6364&idArt=3650782) | 15 |
| France | 310 | - | - | [Y](http://www.sante-sports.gouv.fr/dossiers/sante/grippe-A-H1N1/informations-grand-public/conseils-pour-voyageurs.html) | - | [1 May](http://72.14.203.132/translate_c?hl=en&sl=fr&tl=en&u=http://www.sante-sports.gouv.fr/actualite-presse/presse-sante/communiques/nouveau-virus-grippe-h1n1-information-professionnels-sante.html&prev=hp&rurl=translate.google.com&usg=ALkJrhgKneSuwY-K1zer-IfsU9rmRfb_Ww) | [13 June](http://www.eurosurveillance.org/ViewArticle.aspx?ArticleId=19265) | 43 |
| Guatemala | 286 | - | - | [Y](http://uk.reuters.com/article/idUKN27528938) | - | [5 May](http://new.paho.org/hq/index.php?option=com_content&task=view&id=1312&Itemid=1206) | [1 June](http://en.trend.az/news/world/wnews/1479085.html) | 27 |
| Costa Rica | 277 | - | - | - | - | [29 April](http://www.internationalsos.com/pandemicpreparedness/CountryLevel.aspx?languageID=ENG&countryID=122&catID=27) | [21 May](http://www.nacion.com/ln_ee/2009/mayo/22/pais1972182.html) | 22 |
| Venezuela | 206 | - | - | [Y](http://www.minci.gob.ve/noticias/1/189578/confirmados_12_casos.html) | - | [28 May](http://www.rnv.gov.ve/noticias/?act=ST&f=2&t=98548) | ? |  |
| Ecuador | 204 | - | [Y](http://www.turismo.gov.ec/index.php?option=com_content&task=view&id=1216&Itemid=43) | [Y](http://www.msp.gov.ec/index.php?option=com_content&task=view&id=519&Itemid=84) | - | [15 May](http://new.paho.org/hq/index.php?option=com_content&task=view&id=1373&Itemid=1206) | ? |  |
| Republic of Korea | 202 | - | - | [Y](http://www.stripes.com/article.asp?section=104&article=62345) | [Y](http://www.stripes.com/article.asp?section=104&article=62345) | [2 May](http://www.reliefweb.int/rw/rwb.nsf/db900sid/LSGZ-7RQJDD?OpenDocument&rc=3&emid=EP-2009-000084-MEX) | ? |  |
| Viet Nam | 181 | [Y](https://www.osac.gov/Reports/report.cfm?contentID=102049) | [Y](https://www.osac.gov/Reports/report.cfm?contentID=102049) | - | [Y](https://www.osac.gov/Reports/report.cfm?contentID=102049) | [31 May](https://www.osac.gov/Reports/report.cfm?contentID=102953) | ? |  |
| Greece | 151 | - | - | - | [Y](http://www.cretegazette.com/2009-06/crete-airports-swine-flu.php) | [19 May](http://www.euro.who.int/influenza/AH1N1/20090520_2) | [26 May](http://www.eurosurveillance.org/ViewArticle.aspx?ArticleId=19226) | 7 |
| India | 129 | - | [Y](http://www.centreforaviation.com/news/2009/05/12/confirmed-swine-flu-cases-still-growing-24-countries-now-affected---latest-aviation-updates/page1) | - | [Y](http://www.internationalsos.com/PandemicPreparedness/SubCatLevel.aspx?li=5&languageID=ENG&subCatID=87) | [16 May](http://mohfw.nic.in/Status_as_on_16.5.2009_at_4.30_p.m.doc) | ? |  |
| Honduras | 123 | - | - | - | - | [21 May](http://new.paho.org/hq/index.php?option=com_content&task=view&id=1408&Itemid=1206) | [21 May](http://www.internationalsos.com/pandemicpreparedness/CountryLevel.aspx?languageID=ENG&countryID=31&catID=27) | 0 |
| Saudi Arabia | 114 | - | - | [Y](http://www.centreforaviation.com/news/2009/05/12/confirmed-swine-flu-cases-still-growing-24-countries-now-affected---latest-aviation-updates/page1) | [Y](http://www.meditherm.com/assets/Meditherm news release 04.30 (1).doc) | [3 June](http://news.xinhuanet.com/english/2009-06/03/content_11481705.htm) | ? |  |
| Malaysia | 112 | - | [Y](http://www.infosihat.gov.my/menuutama/Wabak/SwineFlu/Technical Presentation/Flu A H1N1 - Preventive & Control Actions (13Mei09).pdf) | [Y](http://www.google.com/url?sa=t&source=web&ct=res&cd=9&url=http%3A%2F%2Fwww.moh.gov.my%2FMohPortal%2FDownloadServlet%3Fid%3D3221%26type%3D2&ei=mdSISp6_NdCJkQWJ3rypCA&usg=AFQjCNHAnQIuvPVs4UvT8lvVMCMcEn0WMA&sig2=zzaFedxd2tdedNJXnIC19Q) | [Y](http://www.infosihat.gov.my/menuutama/Wabak/SwineFlu/Technical Presentation/Flu A H1N1 - Preventive & Control Actions (13Mei09).pdf) | [15 May](http://thestar.com.my/news/story.asp?file=/2009/5/15/nation/20090515141134) | [17 June](http://thestar.com.my/news/story.asp?file=/2009/6/17/nation/20090617171933&sec=nation) | 33 |
| Cyprus | 109 | - | - | - | - | [30 May](http://www.reuters.com/article/latestCrisis/idUSLU583769) | [25 June](http://news.xinhuanet.com/english/2009-06/30/content_11623253.htm) | 26 |
| Dominican Republic | 108 | - | - | - | [Y](http://www.meditherm.com/assets/Meditherm news release 04.30 (1).doc) | [27 May](http://new.paho.org/hq/index.php?option=com_content&task=view&id=1421&Itemid=1206) | [31 May](http://elnuevodiario.com.do/app/article.aspx?id=154014) | 4 |

* Entry screening methods used included the following:

1. Medical checks were carried out by some countries on an ad-hoc basis, targeting travelers (or entire flights) arriving from specific countries or regions.

2. Health declaration forms were used by some countries to screen arriving travelers. It can be a serious offence to provide false information on a health declaration form.

3. In some countries, alert health officials surveilled arriving travelers for specific symptoms (e.g. cough).

4. Some countries used thermal scanners to identify arriving travelers with high body temperature.

Typically all of the above measures were combined with medical isolation of suspected or confirmed cases, and quarantine of close contacts (for example family members, airplane passengers in surrounding seats).

^†^ China here indicates mainland China only, whereas the WHO website listing for China includes cases reported by mainland China and the Hong Kong and Macau Special Administrative Regions. These regions have separate border controls. Hong Kong is listed separately above, while Macau is not listed in this table as it had only reported 60 cases by July 6.

"-" indicates that a screening method was not used.

"?" indicates that the first untraced case was not reported.
